# Genetic variant in vitamin D-binding protein is associated with metabolic syndrome and lower 25-hydroxyvitamin D levels in polycystic ovary syndrome: A cross-sectional study

**DOI:** 10.1371/journal.pone.0173695

**Published:** 2017-03-09

**Authors:** Betânia Rodrigues Santos, Sheila Bünecker Lecke, Poli Mara Spritzer

**Affiliations:** 1 Gynecological Endocrinology Unit, Division of Endocrinology, Hospital de Clínicas de Porto Alegre, Porto Alegre, Rio Grande do Sul, Brazil; 2 Laboratory of Molecular Endocrinology, Department of Physiology, Federal University of Rio Grande do Sul, Porto Alegre, Rio Grande do Sul, Brazil; 3 Department of Diagnostic Methods, Federal University of Health Sciences of Porto Alegre, Porto Alegre, Rio Grande do Sul, Brazil; Peking University Third Hospital, CHINA

## Abstract

Vitamin D deficiency has been related to metabolic syndrome (MetS) in polycystic ovary syndrome (PCOS). The vitamin D-binding protein (DBP) is the main protein involved in vitamin D transport. Two single-nucleotide polymorphisms (SNPs) of the *DBP* gene, rs4588 and rs7041, have been associated with low circulating levels of 25-hydroxyvitamin D [25(OH)D] in various populations, but not in women with PCOS. Therefore, we determined the genotype and haplotype distribution of *DBP* gene polymorphisms and investigated the associations between these genetic variants and their haplotypes with PCOS, MetS, and 25(OH)D levels in women with PCOS and controls from the South of Brazil. The sample included 291 women (191 with PCOS and 100 controls). All participants were genotyped for polymorphisms rs2282679, rs4588, and rs7041. Serum 25(OH)D levels were determined in a subset of 102 participants. Women with PCOS were younger and had significantly higher body mass index, blood pressure, and insulin resistance than the control group (p<0.05). The prevalence of MetS in PCOS and controls was 26.5% and 4.8% respectively. Levels of 25(OH)D were lower in PCOS women with MetS, even after adjustment for age (p = 0.033). No associations were observed between PCOS and the polymorphisms or their haplotypes. A higher frequency of genotype TT of rs7041 was found in PCOS participants with MetS (OR: 2.21, 95%CI:1.08–4.52; p = 0.027). This same genotype was associated with lower 25(OH)D levels in both PCOS and control women (OR: 4.40, 95%CI:1.62–12.00; p = 0.002). In conclusion, these findings indicate that *DBP* gene polymorphisms and their haplotypes are not directly associated with PCOS. In contrast, the TT genotype of SNP rs7041 was associated with MetS in PCOS women, and with lower 25(OH)D levels in both PCOS and control groups.

## Introduction

Polycystic ovary syndrome (PCOS), a heterogeneous disease characterized by chronic anovulation and manifestations of hyperandrogenism [1, 2], affects between 9% and 18% of women of reproductive age, depending on diagnostic criteria [1–3]. Women with PCOS suffer from metabolic abnormalities, including insulin resistance (IR), obesity, and metabolic syndrome (MetS) [4–6]. Accumulating evidence suggests that vitamin D deficiency is associated with IR and MetS in PCOS [7]. Vitamin D deficiency may also be linked to central obesity, lipid profile, and body mass index (BMI) in these women [7–13].

Both the vitamin D receptor (VDR) and the vitamin D-binding protein (DBP) play a key role in vitamin D metabolism. VDR is expressed in many tissues and organs, such as those involved in calcium homeostasis, glucose metabolism, and reproduction [14], whereas DBP is the main protein involved in vitamin D transport [15, 16]. Two well-studied single-nucleotide polymorphisms (SNPs) of the *DBP* gene, rs4588 and rs7041, have been previously shown to be strongly associated with low circulating 25-hydroxyvitamin D [25(OH)D] levels in genome-wide association studies [17, 18] and in various populations [19–24].

Although there have been reports about VDR polymorphisms in PCOS [25–27], few studies have focused on *DBP* gene polymorphisms in women with PCOS or androgen excess. The single work published to date showed similar genotype frequencies of SNP rs2282679 in PCOS and controls [25]. In other populations, polymorphisms of *DBP* gene were associated with several endocrine and metabolic parameters [28–30].

Therefore, the aim of the present study was to compare the frequency of SNPs rs2282679, rs4588, and rs7041 of the *DBP* gene and their haplotypes in women with PCOS and healthy controls with regular ovulatory cycles from Southern Brazil. We also aimed to investigate whether these genetic variants are related to MetS and 25(OH)D levels in PCOS women.

## Materials and methods

### Patients

Participants were enrolled by advertisement in the local media. The advertisement called for women of reproductive age with excess hair (hirsutism) and irregular menses and for volunteers without hirsutism and with regular menses. The study population comprised 291 women: 191 patients with PCOS and 100 non-hirsute women with regular ovulatory cycles (confirmed by progesterone levels higher than 3.8 ng/mL). Diagnostic investigation was performed for all enrolled participants at a university hospital (Hospital de Clínicas de Porto Alegre, state of Rio Grande do Sul).

Rotterdam criteria were used for the diagnosis of PCOS, which was defined in the presence of two out of three of the following traits: 1) oligo/amenorrhea and/or chronic anovulation (≤9 cycles/year and/or luteal phase progesterone ≤3.8 ng/mL), 2) clinical and/or biochemical hyperandrogenism, and 3) polycystic ovary appearance on ultrasound examination. Exclusion criteria were presence of hyperandrogenic disorders, having used drugs known to interfere with hormone levels (such as oral contraceptive pills, antiandrogens, progestins, metformin, fibrates, or statins) for 3 or more months before the study, pregnancy, liver disease, or kidney disease.

Approval for this study was obtained from the Institutional Review Board at Hospital de Clínicas de Porto Alegre. Written informed consent was obtained from all participants.

### Study protocol

Anthropometric measurements included BMI and waist circumference (WC). Blood pressure (BP) was measured twice after a 10-minute rest [4, 5, 31–33].

Hirsutism was defined as a modified Ferriman-Gallwey score [34] ≥8. Homeostasis model assessment index to estimate insulin resistance (HOMA-IR) was calculated by multiplying insulin (mIU/mL) by glucose (mmol/L) and dividing this product by 22.5 [35]. Joint Scientific Statement criteria were used to define MetS [36].

### Laboratory measurements

Blood samples for determination of hormone levels were drawn from an antecubital vein after a 12-h overnight fast, between 8:00 and 10:00 am. Samples were obtained between the 2nd and 10th days of the cycle, or on any day in amenorrheic women. Blood samples were also collected for genomic DNA extraction.

Total cholesterol (TC), high-density lipoprotein cholesterol (HDL-c), triglycerides (TG), and fasting glucose levels were determined by colorimetric-enzymatic methods (Siemens Advia 1650, Deerfield, USA). Low-density lipoprotein cholesterol (LDL-c) was estimated indirectly with the Friedewald formula [37]. Total testosterone and insulin levels were measured by chemiluminescence (Siemens Advia Centaur XP, Deerfield, USA). Serum 25(OH)D levels were measured in a subset of 102 women by a chemiluminescence assay (Liaison, DiaSorin, Saluggia, Italy) with sensitivity of ≤4.0 ng/mL and intra- and inter-assay CV of 7.7% and 10.9%, respectively.

### Genotype analysis

Genomic DNA was extracted from peripheral blood leukocytes [38]. DNA samples were diluted to 2 ng/mL. Duplicate measurements were performed in 10% of the samples to assess the internal quality of genotype data. Molecular genotyping for rs4588 (substitution of C for A), rs7041 (substitution of T for G), and rs2282679 (substitution of A for C) was performed through real-time polymerase chain reaction (PCR) (7500 Fast Real-Time Polymerase Chain Reaction System, Applied Biosystems, California, USA), using the allelic discrimination assay with TaqMan MGB primers and probes (Applied Biosystems, California, USA).

### Statistical analysis

Data distribution was assessed by Kolmogorov-Smirnov test and descriptive statistics. Results are expressed as mean ± standard deviation (SD) for normally distributed variables, as median and interquartile range for variables with a non-Gaussian distribution, or as absolute numbers and percentages. Non-Gaussian variables were log-transformed for statistical analysis and back-transformed into their original units for reporting. Unpaired two-tailed Student’s t-test was used to compare group means. Pearson’s chi-square test (χ^2^) was applied to test categorical variables and the agreement of genotype frequencies with Hardy-Weinberg equilibrium.

Haplotypes were constructed from the combination of the rs4588 and rs7041 polymorphisms. Lewontin’s D’ statistic for linkage disequilibrium was calculated for each pair of polymorphisms. Linkage disequilibrium was inferred using the Phase 2.1.1 software [39], which employs Bayesian statistics. This software was also used to compare haplotype frequencies in PCOS and control women by permutation testing. Data were considered as statistically significant at p<0.05. The Statistical Package for the Social Sciences 18 (SPSS, Chicago, IL) was used for analyses.

## Results

Participants were mostly white (93.9%), and some (6.1%) had mixed African and European ancestry. Clinical characteristics of the sample are shown in Table 1. The mean age of PCOS and control participants was 22.89±6.66 and 25.18±7.72 years respectively (p = 0.013, Student’s t test). As expected, women with PCOS had significantly higher BMI, WC, BP, HOMA-IR, triglycerides, Ferriman-Gallwey score, and total testosterone, as well as lower HDL-c than controls (p<0.05 for all variables, Student’s t test). MetS was more frequent in PCOS participants (p<0.001, Pearson’s χ^2^ test).

**Table 1:** Clinical and biochemical profile of PCOS and control participants.

Variable	Controls (n = 100)	PCOS (n = 191)	p
BMI (kg/m^2^)	27.04±6.09	29.70±6.40	0.001
WC (cm)	78.04±11.51	89.23±15.08	<0.001
Systolic BP (mmHg)	109.52±12.90	121.10±15.50	<0.001
Diastolic BP (mmHg)	70.83±9.39	78.06±11.53	<0.001
Fasting glucose (mg/dL)	88.53±7.57	88.89±15.30	0.797
HOMA-IR	2.18 (1.42–3.14)	3.52 (1.96–6.36)	<0.001
TC (mg/dL)	170.11±30.72	174.69±38.31	0.290
HDL-c (mg/dL)	52.84±12.28	48.85±10.87	0.007
LDL-c (mg/dL)	101.70±26.30	104.51±31.82	0.443
Triglycerides (mg/dL)	66.00 (50.00–99.00)	89.00 (62.00–131.00)	<0.001
Ferriman-Gallwey	2.19±2.10	15.55±6.11	<0.001
TT (ng/mL)	0.55 (0.42–0.64)	0.82 (0.62–1.11)	<0.001
Metabolic syndrome	4.8%	26.5%	<0.001

Data are expressed as mean ± SD, median (interquartile range) (Student’s t test), or percentage. p value by Pearson’s χ^2^ test. PCOS: polycystic ovary syndrome; BMI: body mass index; WC: waist circumference; BP: blood pressure; HOMA-IR: homeostasis model assessment index to estimate insulin resistance; TC: total cholesterol, HDL-c: high-density lipoprotein cholesterol, LDL-c: low-density lipoprotein cholesterol; TT: total testosterone.

Serum 25(OH)D levels were measured in a subset of participants (54 PCOS and 48 controls) who had an extra serum aliquot available for this measurement. Participants with and without measured 25(OH)D levels were similar regarding BMI (p = 0.545), HOMA-IR (p = 0.110), and presence of MetS (p = 0.540).

**Fig 1:**
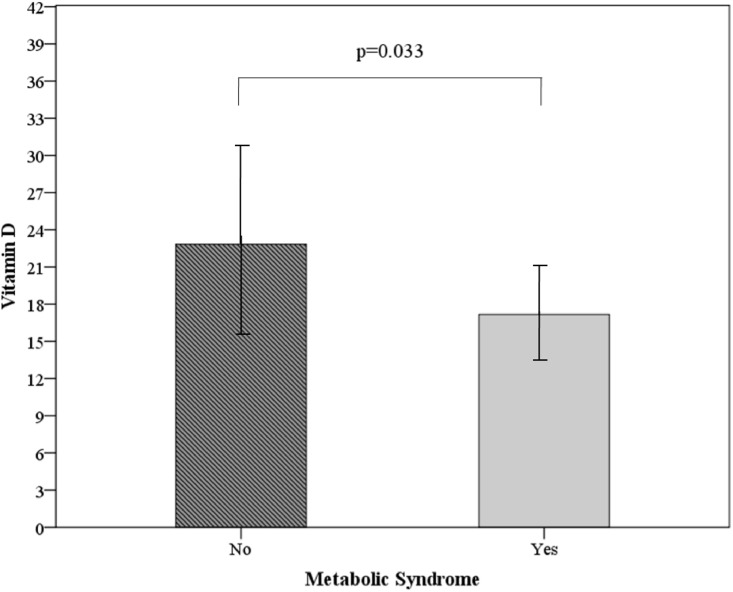
25(OH)D levels according to presence of the metabolic syndrome in 54 PCOS participants. Data are expressed as mean ± SD. p value by Student’s t test, adjusted for age. 25(OH)D: 25-hydroxyvitamin D.

The mean 25(OH)D concentration in the subset was 21.48±7.25 ng/mL. Only 12.7% of this subset had adequate circulating levels of 25(OH)D (≥ 30 ng/mL). In 45.1%, 25(OH)D levels were insufficient (20–29 ng/mL), and 42.2% had vitamin D deficiency (<20 ng/mL). Sufficient vitamin D status was similar in the PCOS and control women included in the subset (14.8% vs. 10.4% respectively). Also, 25(OH)D values were similarly low in both subset groups (21.50±6.90, controls and 21.47±7.61, PCOS; p = 0.985). A separate analysis of the subset PCOS group revealed lower vitamin D levels in women with MetS (p = 0.018, Student’s t test), even after adjustment for age (p = 0.033) (Fig 1).

Regarding *DBP* gene polymorphisms, all three SNPs were in Hardy-Weinberg equilibrium in the PCOS and control groups. Only three, two, and one participants were not genotyped for SNP rs2282679, rs7041, and rs4588 respectively. The genotype and allele frequencies of DBP gene variants are presented in Table 2. The genotype and allele distribution of all three polymorphisms was similar in PCOS and controls.

**Table 2:** Genotype, allele, and haplotype frequencies of *DBP* gene variants in PCOS and control women.

SNP	Controls n (%)	PCOS n (%)	p
rs2282679			
AA	61 (61%)	103 (55%)	0.360
AC	33 (33%)	65 (35%)	
CC	6 (6%)	20 (10%)	
A	155 (77%)	271 (72%)	0.159
C	45 (23%)	105 (27%)	
rs4588			
CC	61 (61%)	104 (55%)	0.479
CA	32 (32%)	66 (35%)	
AA	7 (7%)	20 (10%)	
C	154 (77%)	274 (72%)	0.203
A	46 (23%)	106 (28%)	
rs7041			
TT	24 (24%)	47 (25%)	0.911
TG	45 (45%)	88 (47%)	
GG	31 (31%)	54 (28%)	
T	93 (47%)	182 (48%)	0.706
G	107 (53%)	196 (52%)	
Haplotypes			
CG CG + CT CG	54 (54%)	94 (49.3%)	0.740
CG AT + CT CT	29 (29%)	61 (31.9%)	
CT AT + AT AT	17 (17%)	36 (18.8%)	

Data are expressed as percentage. p value by Pearson’s χ^2^ test. The rs4588-rs7041 haplotype is grouped by presence of the risk haplotype.

Polymorphism rs4588 (C→A) was in linkage disequilibrium with rs7041 (T→G) (|D’| = 1; r^2^ = 0.44). Three haplotypes were inferred in the sample: CT, CG, and AT, formally called GC-1f, GC-1s, and GC-2 respectively. The first letter of each haplotype refers to SNP rs4588 and the second to SNP rs7041. Haplotype frequencies were 0.21 for CT, 0.53 for CG, and 0.26 for AT. The three common haplotype variants (CT, CG, and AT) formed six diplotypes: CT-CT, CT-CG, CG-CG, CG-AT, CT-AT, and AT-AT, with frequencies of 0.06, 0.21, 0.30, 0.25, 0.09, and 0.09 respectively.

Taking into consideration the results of individual polymorphism analyses and previous data from the literature [19–24], AT was regarded as the risk haplotype and CG as the protective haplotype against lower 25(OH)D concentrations. Therefore, we grouped haplotype combinations accordingly: CG CG + CT CG, CG AT + CT CT, and CT AT + AT AT. Haplotype frequencies were similar in PCOS and control groups (Table 2).

**Fig 2:**
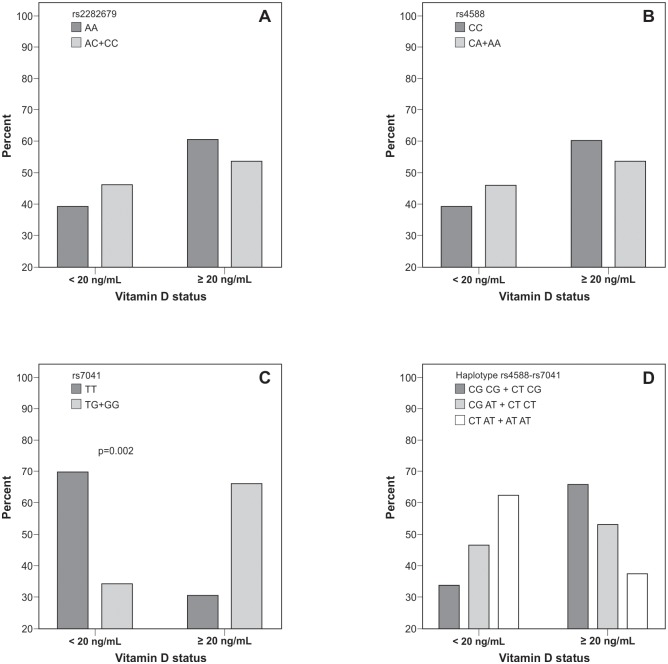
Genotype and haplotype distribution of *DBP* gene according to vitamin D status in participants with and without PCOS. Data are expressed as percentages. p value by Pearson’s χ^2^ test. DBP: vitamin D-binding protein. **A**: rs2282679 (p = 0.542). **B**: rs4588 (p = 0.542). **C**: rs7041 (p = 0.002). **D**: haplotype rs4588-rs7041, grouped according to the presence of the risk haplotype (p = 0.104).

When all participants were analyzed (PCOS and controls), 69.6% of the women carrying the TT genotype of rs7041 were deficient in vitamin D (<20 ng/mL) and 30.4% presented vitamin D levels ≥20 ng/mL (OR: 4.402, 95% CI: 1.62–12.00; p = 0.002, Pearson’s χ^2^ test). Conversely, vitamin D status was similar for polymorphisms rs2282679 and rs4588 and haplotype variants (Fig 2).

**Fig 3:**
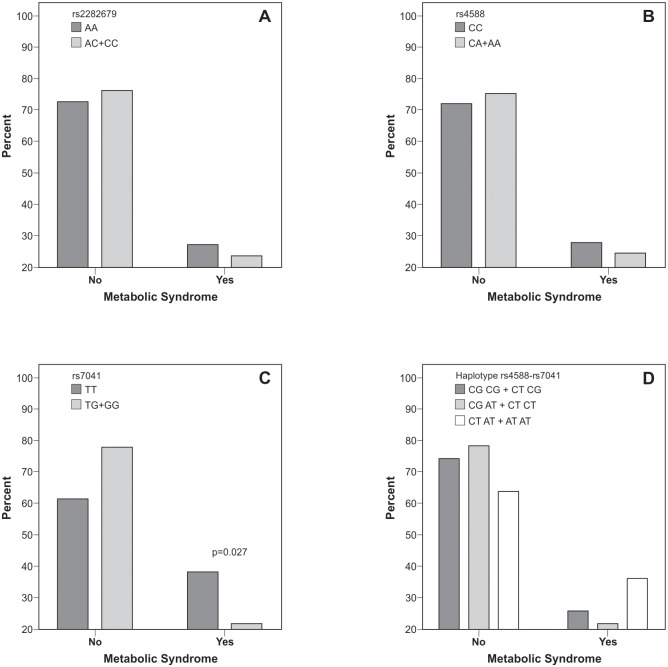
Genotype and haplotype distribution of *DBP* gene according to metabolic syndrome in PCOS participants. Data are expressed as percentage. p value by Pearson’s χ^2^ test. DBP: vitamin D-binding protein; PCOS: polycystic ovary syndrome. **A**: rs2282679 (p = 0.593). **B**: rs4588 (p = 0.613). **C**: rs7041 (p = 0.027). **D**: haplotype rs4588-rs7041, grouped according to the presence of the risk haplotype (p = 0.294).

Within the PCOS group, a higher frequency of the TT genotype of rs7041 was observed in the presence of MetS (OR: 2.21, 95% CI: 1.08–4.52; p = 0.027, Pearson χ^2^ test). This was not observed for rs2282679, rs4588, and the rs4588-rs7041 haplotype, whose frequencies were similar in PCOS participants with or without MetS (Fig 3).

## Discussion

In the present study, the frequency of SNPs rs2282679, rs4588, and rs7041 of the *DBP* gene and their haplotypes was similar in women with PCOS and healthy controls with regular ovulatory cycles from Southern Brazil. In turn, polymorphism rs7041 in the *DBP* gene was related to lower 25(OH)D levels in the overall group and to MetS in PCOS—women with PCOS carrying the TT genotype of rs7041 were twice as likely to present MetS. To the best of our knowledge, this is the first report to show an association between MetS and polymorphism rs7041 in a PCOS population.

Our results also show that 25(OH)D levels were lower in PCOS participants with MetS. A recent meta-analysis has reported that vitamin D levels are indeed related to metabolic and hormonal disturbances in PCOS women. In that study, women with PCOS and vitamin D deficiency were more likely to have dysglycemia compared to those without vitamin D deficiency [11]. In addition, a study comparing PCOS women and controls showed that vitamin D levels were lower in participants with both PCOS and MetS compared to those with PCOS but without MetS. Furthermore, vitamin D levels decreased as the number of risk factors for MetS increased [12]. Moreover, in PCOS women, lower vitamin D levels have been correlated with clinical traits, insulin resistance measures, and lipid profile [8, 12, 13].

Only a few studies have assessed SNPs rs4588 and rs7041 in relation to metabolic parameters in other populations. Some studies have shown an association of polymorphisms in exon 11 with circulating levels of insulin and HOMA-IR [28] and with glucose levels [29] in non-diabetic individuals. Nevertheless, these polymorphisms have not been associated with type 2 diabetes [29, 30].

Regarding polymorphisms rs4588 and rs7041 and vitamin D levels, one study reported no interaction between 25(OH)D and SNPs of *DBP* [40], while another showed a marginal interaction of 25(OH)D deficiency with rs7041 in white subjects [41]. In adult and elderly populations, two studies with Chinese participants have shown that both SNPs rs4588 and rs7041, as well as the AT-AT haplotype, were related to lower 25(OH)D levels [22, 23]. Similar results were reported in Canadian young adults [20] and elderly Caucasians [42]. Finally, lower 25(OH)D concentrations have been observed in premenopausal white women [19] and early postmenopausal women [43] carrying the AA genotype of rs4588 and the TT genotype of rs7041. Taken together, data from the literature and the present results showing an association between the TT genotype of rs7041 and vitamin D deficiency support the hypothesis that this *DBP* gene variant is related to 25(OH)D concentrations. In addition, the main finding of this study—that polymorphism rs7041 in the *DBP* gene is related to metabolic syndrome in PCOS and to lower 25(OH)D levels in the overall group—might signal a genetic link between metabolic disturbances in PCOS and low vitamin D levels.

In a previous study with 545 Austrian women with PCOS aged 16–45 years, no higher risk of PCOS was found in association with genotypes of rs2282679. However, anthropometric variables and lipid profile differed significantly among rs2282679 genotypes [25]. In contrast, we did not find associations between MetS and rs2282679 genotypes. Ethnic differences between the two populations, as well as the older age of participants in the study by Wehr et al. [25], may explain this disagreement.

The *DBP* gene encodes a multifunctional plasma transport protein, DBP, also known as a group-specific component, synthesized in the liver. DBP binds and transports vitamin D and its metabolites to target tissues. DBP exerts important biological functions, including the binding of mainly monounsaturated and saturated fatty acids [15]. A link between obesity and vitamin D has been described in PCOS [8] and in other populations [44–49]. The relationship with DBP is, however, less clear. In elderly men, a positive relationship has been described between DBP concentrations and BMI [50]. However, this association has not been confirmed in women aged 18–44 years [51]. Overall, the mechanisms underlying this association are still unknown, but deserve further investigation.

It should also be noted that polymorphisms rs4588 and rs7041, which are located in exon 11, were in linkage disequilibrium, making it difficult to discern the best single SNP surrogate to detect genetic variability for this region. Indeed, rs4588 and rs7041, described as having an association with 25(OH)D levels, were in linkage disequilibrium in a healthy population of girls from Southern Brazil [24].

One strength of our study is the focus on a less well represented ethnic group, PCOS women from Southern Brazil. Conversely, a limitation was the relatively small sample size of 291 participants, which does not allow supplemental analyses. However, the effect sizes observed in our sample are similar to those reported in other PCOS populations. Another limitation was the lack of data on DBP levels of participants. Nevertheless, *DBP* gene polymorphism seems to have no effect on the relationship between 25(OH)D and parathyroid hormone in infants and toddlers [21]. Other possible limitations are the lack of data on dietary vitamin D intake and daily sun exposure, even though it is well recognized that, below a latitude of approximately 35°, UVB radiation is sufficient for year-round vitamin D synthesis [52]; furthermore, the season of blood collection seems not to interfere with vitamin D levels, as we have previously shown in another group living in the same region as the present sample [24]. While DBP measurements were not available in the present study, previous studies have shown that DBP levels are associated with *DBP* gene polymorphisms [43, 53] and positively correlated with 25(OH)D levels [21].

## Conclusions

The present study is the first to describe that genotype TT of SNP rs7041 is associated with MetS in PCOS and with lower 25(OH)D levels in both PCOS and healthy controls with regular ovulatory cycles. Our results indicate that polymorphisms rs2282679, rs4588, and rs7041 of the *DBP* gene, as well as their haplotypes, are not related to PCOS in southern Brazilian women. Further studies in PCOS populations of different ethnicities are needed to confirm these findings.
